# Versatile Polypeptide-Functionalized Plasmonic Paper as Synergistic Biocompatible and Antimicrobial Nanoplatform

**DOI:** 10.3390/molecules25143182

**Published:** 2020-07-13

**Authors:** Leopold Tie, Mina Răileanu, Mihaela Bacalum, Irina Codita, Ștefania Mădălina Negrea, Costin Ștefan Caracoti, Elena-Carmina Drăgulescu, Andreea Campu, Simion Astilean, Monica Focsan

**Affiliations:** 1 Nanobiophotonics and Laser Microspectroscopy Center, Interdisciplinary Research Institute on Bio-Nano-Sciences, Babes-Bolyai University, Treboniu Laurean No.42, 400271 Cluj-Napoca, Romania; tiebidjeleopold@gmail.com (L.T.); andreea.campu@gmail.com (A.C.); simion.astilean@phys.ubbcluj.ro (S.A.); 2 Biomolecular Physics Department, Faculty of Physics, Babes-Bolyai University, M Kogalniceanu No. 1, 400084 Cluj-Napoca, Romania; 3 Department of Life and Environmental Physics, Horia Hulubei National Institute of Physics and Nuclear Engineering, 30 Reactorului Street, 077125 Magurele, Romania; mina.raileanu@nipne.ro (M.R.); bmihaela@nipne.ro (M.B.); 4 Department of Electricity, Solid State and Biophysics, Faculty of Physics, University of Bucharest, 077125 Măgurele, Romania; 5 Cantacuzino National Medical-Military Institute for Research-Development, Splaiul Independenței 103, 050096 Bucharest, Romania; adirina_2005@yahoo.com (I.C.); madalinanegreas@gmail.com (S.M.N.); caracoti.costin@gmail.com (C.S.C.); elena_dragulescu@yahoo.com (E.-C.D.)

**Keywords:** gold nanospheres, paper platform, antimicrobial peptides, biocompatibility, antimicrobial activity, *Staphylococcus aureus*, *Escherichia coli*

## Abstract

Nowadays, thanks to nanotechnological progress, which itself guides us more and more closely toward not only the efficient design of innovative nanomaterials or nanostructures, but to the improvement of their functionality, we benefit from an important asset in the battle against pathogenic illnesses. Herein, we report a versatile biocompatible plasmonic nanoplatform based on a Whatman paper incorporating positively-charged gold nanospherical particles via the immersion approach. The morphological characterization of the as-engineered-plasmonic paper was examined by SEM (scanning electron microscopy) and HRTEM (high-resolution transmission electron microscopy) investigations, while its surface chemical modification with a synthetic polypeptide, specifically RRWHRWWRR-NH2 (P2), was proved by monitoring the plasmonic response of loaded gold nanospheres and the emission signal of P2 via fluorescence spectroscopy. The as-functionalized plasmonic paper is non-cytotoxic towards BJ fibroblast human cells at bactericidal concentrations. Finally, the antimicrobial activity of the P2-functionalized plasmonic paper on both planktonic bacteria and biofilms was tested against two reference strains: Gram-positive Bacteria, i.e., *Staphylococcus aureus* and the Gram-negative Bacteria, i.e., *Escherichia coli*, determining microbial inhibition of up to 100% for planktonic bacteria. In line with the above presented nanoplatform’s proper design, followed by their functionalization with active antimicrobial peptides, new roads can be open for determining antibiotic-free treatments against different relevant pathogens.

## 1. Introduction

In present times, bacterial drug resistance can lead to serious health problems worldwide due to the long-term use of traditional antibiotics resulting in bacteria becoming immune to treatment [1]. Finding new antibiotics is becoming more and more difficult, and, therefore, new types of antibacterial compounds or novel innovative therapeutic approaches should be designed to solve this growing medical problem, being one of the top priorities for 2020, according to the World Health Organization (WHO). In this context, metal nanoparticles, based on their physical and chemical properties, have been recently proposed as versatile nanoplatforms of high research interest [2]. In particular, although silver nanoparticles were proved to be very effective against bacteria strains [3], presenting antimicrobial activity both in the dark and under illumination [4,5], an important concern is still represented by their induced cytotoxicity, thus limiting their implementation in biological applications. However, certain innovative safety systems were designed by D’Agostino et al. based on triangular silver nanoparticles which successfully proved their ability to act as powerful antimicrobial agents, by simply merging the controlled release of a very low concentration of silver ions in water with the hyperthermia effect generated by the photo-thermal activation under NIR irradiation at 808 nm. More interesting, the long-term antibacterial protection can be reinforced as needed by a fast, localized photothermal activation of the anisotropic-shaped silver nanoparticles, consequently ensuring an additional elimination of the bacterial cells [6,7]. On the other hand, gold nanoparticles exhibit low toxicity [8,9]; however, sometimes their intrinsic size and shape-dependent antibacterial activity seems to be insufficient. To overcome this problem, the surface modification of the plasmonic nanoparticles could be an answer, through use of controlled grafting of different charged molecules facilitating specific binding to the bacterial membrane, and, consequently, generating an increase of their antibacterial activity [10]. Taking profit of the tunable sizes and shapes, as well as their easy surface functionalization, differently-functionalized gold nanoparticles started to be widely investigated as antibacterial, antifungal, antibiotic film platforms [11]. For example, Scaiano’s group, using amoxicillin coating of the gold nanoparticles surface, proved synergistic antimicrobial activity upon light irradiation against sensitive and antibiotic-resistant *Staphylococcus aureus* [12], while by employing lignin as a natural reducing and capping agent, they demonstrated that these formed non-toxic nanocomposites are able to act as bacteriostatic agents against bacterial biofilms [13]. However, by grafting onto the gold nanoparticles’ surface antimicrobial peptides (AMPs) [14], well-known as natural antibiotics, it would be possible to significantly increase their antibacterial activity. In particular, AMPs are host defense peptides, most of them being cationic (positively charged, and thus the affinity for the negatively charged bacterial membrane is increased) and amphiphilic (hydrophilic and hydrophobic) α-helical peptide molecules. The negatively-charged membrane permeability is a well-accepted mechanism to describe the action of the cationic AMPs [15], making them well suited for medical applications. Specifically, the AMPs first attach to the bacterial membrane through electrostatic interactions and is followed by the membrane disruption (due to the hydrophobic amino-acids), which is realized through three mechanisms: barrel-stave pore, toroidal pore or carpet model [16,17].

The free-standing paper structure, as a highly versatile, low-cost and biocompatible nanoplatform, can ensure better control of the cell distribution over the extracellular matrix, considering that this support, due to its porous, flexible and intrinsic three dimensional (3D) scaffolds, is able to mimic in a more realistic manner the in-vivo cell microenvironment [18]. Additionally, paper has already been successfully implemented in a plethora of biological applications, starting as paper-based biosensors [19,20], 3D foldable paper electronics [21], and, more recently, as a cell culture platform [22,23].

In the last decade, the use of nanotechnologies, which deal with the manipulation of nanomaterials and their controlled incorporation into paper platforms for decontamination purposes, has gained much attention. Different approaches have been designed to treat pathogens in real time [24], as well as filter the water in order to clean the water sources. The development of affordable and efficient technological solutions is in great demand to battle bacterial pollution, so that the public can access safe drinking water and sanitation. In this context, Jain et al. developed a highly efficient filter paper, proposing a water-resistant cellulose foam paper with a high wetting strength property imbedded with diverse metal oxide (e.g., copper oxide (CuO), zinc oxide (ZnO), and silver oxide (Ag_2_O)) nanoparticles, which was proved to be effective against different strains of bacteria [25]. Nanoplatforms with antimicrobial properties were also obtained using ZnO and TiO_2_ nanostructures grown on Whatman paper, presenting good results on *S. aureus* bacteria [26]. Therefore, paper, with its interesting large porous microstructure, offers not only the necessary space for cell growth, but also enables the incorporation of different nanomaterials, as well as its modification with different ligands of interest, such as peptides, consequently creating a more comfortable microenvironment for cell growth, and, thus, extending to different innovative applications.

In light of fighting against antibiotic-resistant bacteria, in this paper we propose a new 3D effective antibacterial platform realized in two successive steps: (i) the loading in a controlled and efficient manner of pre-synthesized positively charged cetyltrimethylammonium chloride (CTAC) spherical nanoparticles in colloidal solution onto Whatman paper, as a plasmonic matrix, followed by (ii) its surface functionalization with a synthetic polypeptide, specifically RRWHRWWRR-NH2 (denoted as P2). The gold nanoparticles, as antimicrobial nanomaterials, are used herein due to their many advantages, such as: (i) small size and high surface area; (ii) large contact area with bacteria, allowing the destruction of its permeability; (iii) reduced probability of different bacteria to develop drug-resistance to them, and, finally, (iv) low toxicity to mammalian cells compared to silver nanoparticles [27]. While the successful functionalization of the plasmonic paper was demonstrated by the recorded spectral modifications in the localized surface plasmon resonance (LSPR) band and the emission band of the tryptophan residues before and after the P2 grafting, its biocompatibility was tested against human BJ cells. Then, the antimicrobial activity of the new-obtained platform was firstly evaluated on planktonic bacteria against two reference strains: Gram-positive bacteria, i.e., *Staphylococcus aureus ATCC 12600* and the Gram-negative Bacteria, i.e., *Escherichia coli ATCC 25922*, proving an enhanced synergistic effect of 100% when P2 is grafted onto the nanoplatform compared to the free plasmonic paper. Furthermore, the plasmonic paper significantly reduces the in vitro biofilm formation of *Staphylococcus aureus* up to 79% and *Escherichia coli* up to 24% in contrast to the biofilm growth in the absence of the plasmonic paper. To summarize, our designed peptide-functionalized plasmonic paper can be a promising antimicrobial candidate in the future for treating wounds or skin infections.

## 2. Results and Discussion

### 2.1. Optical and Morphological Characterization of the AuNSs Before and after Their Immobilization onto the Paper Substrate

**Figure 1:**
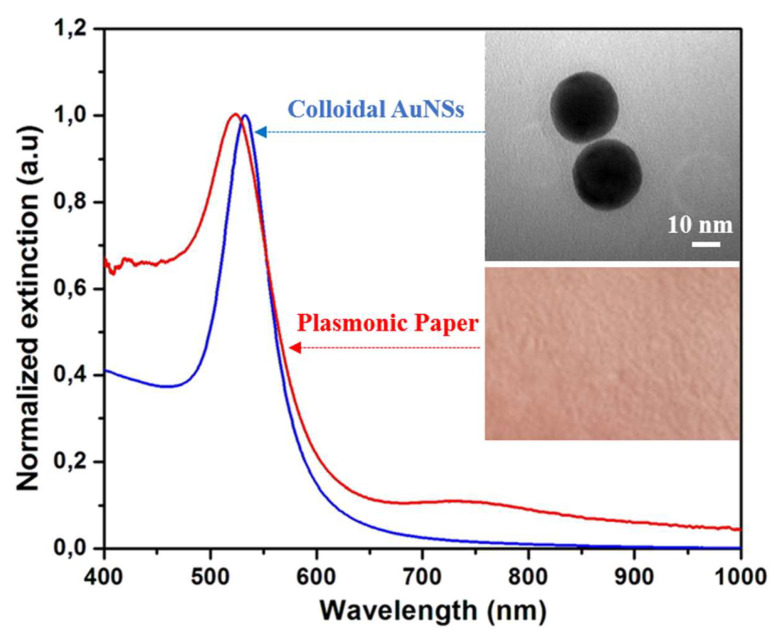
The normalized extinction spectra of the AuNSs before and after their immobilization onto the paper substrate. Inset upper-right: A representative TEM (transmission electron microscope) image of the as-synthesized AuNSs. Inset lower-right: A digital photograph of the paper substrate after the immobilization of the AuNSs.

Prior to the immobilization of the gold nanoparticles, the colloidal AuNSs were characterized in terms of optical properties and morphological features. In this context, the optical response of the as-synthesized nanostructures was recorded using an UV-Vis-NIR spectrophotometer. In Figure 1, the blue spectrum corresponds to the AuNSs in aqueous solution, as expected, they exhibit one LSPR band at 529 nm due to the oscillations of the conduction electrons at the surface of the nanostructures, a phenomenon theoretically described by the Mie theory [28]. The upper-right inset shows a representative TEM image of the as-synthesized AuNSs, thus confirming their spherical shape. Furthermore, by the analysis of the TEM images using the ImageJ toolkit software, the diameter of the nanoparticles was determined to be 35 ± 2 nm.

This result was corroborated with the DLS measurements, which indicate a mean hydrodynamic diameter of 44 nm for the highly monodisperse colloidal AuNSs (data not shown here). Additionally, the surface potential was investigated by zeta potential measurements revealing the positive value of + 51 mV. This particular feature is highly advantageous for the further adsorption of the nanostructures. In fact, the uniform adsorption of the AuNSs onto the paper fibres is due possibly to the electrostatic interaction between the positively charged nanoparticles and paper, which presents a large number of hydroxyl groups that are accessible, in general, for attaching positively charged species [29,30].

The as-synthesized colloidal AuNSs were poured into a Petri dish and the cut paper strips were immersed for 10 min, followed by an additional 10 min drying treatment at 45 °C. After the first immersion, the white Whatman paper became red (Figure 1-Inset lower-right), the colour of the AuNSs in the solution, allowing a first colorimetric confirmation of the successful immobilization of the nanostructures onto the paper substrate. However, the immersion protocol was repeated two more times in order to ensure the highest immobilized AuNSs’ concentration on the paper substrate, without inducing aggregation. Subsequently, the LSPR response of the plasmonic paper was recorded (Figure 1: red spectrum), the optical response of the colloidal nanostructures is well-preserved, indicating the immobilization of individual nanospheres on the cellulose fibres without large scale aggregation. Moreover, the extinction band underwent a blue-shift of 5 nm, which is not surprising given that the LSPR is highly sensitive to the surrounding environment of the nanoparticles, and hence it depends on the refractive index of the medium they are in. By drying the plasmonic paper, the AuNSs are transferred from water (*n* = 1.333) and placed in air (*n* = 1). All of these results confirm the successful immobilization of the AuNSs onto the paper substrate.

**Figure 2:**
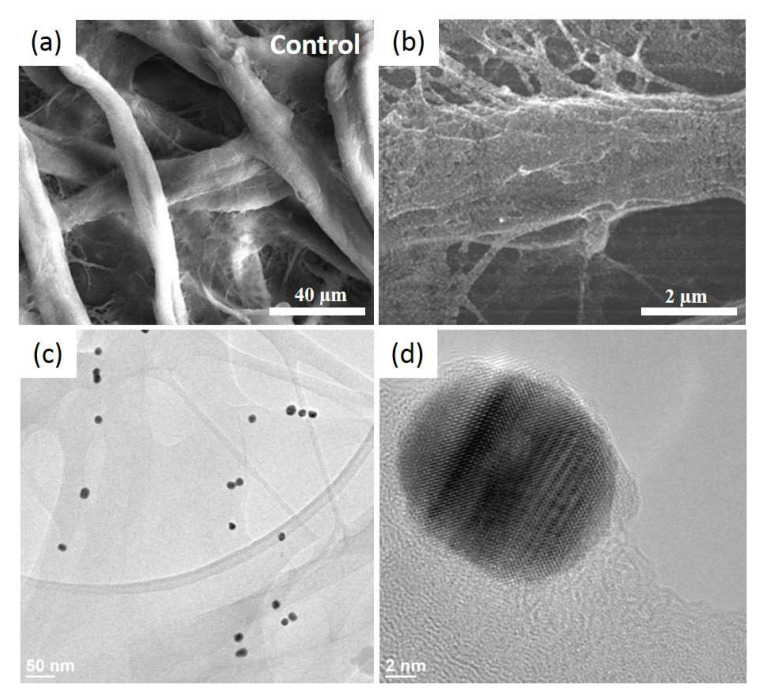
Representative SEM (scanning electron microscope) images of the pristine Whatman paper presenting its interconnected microfibers (**a**) and after the uniform decoration of the paper surface with the AuNSs (bright spots) (**b**), as well as typical HRTEM (high-resolution transmission electron microscope) images at low (**c**) and high resolution (**d**).

Furthermore, the SEM analysis of the plasmonic paper consolidates the obtained optical results. Figure 2a,b present representative SEM images of the cellulose fibres before and after the AuNSs immobilization onto the cellulosic fibres (as white dots), respectively.

The AuNSs are well-adsorbed on the 3D porous structure of the Whatman paper, thanks to the electrostatic interaction between the two opposite surface charges. Additionally, they present a homogenous distribution like a thin film, without large scale aggregation. For a higher magnification, HRTEM was employed to distinguish the individual nanostructures (Figure 2c) by wetting the paper with alcohol and scratching it to obtain a debris, which was then dropped onto a carbon grid and left to dry prior to the analysis. The HRTEM-obtained results are in good agreement with the optical determinations and concluding that, after the immobilization, the AuNSs maintained their shape and size; the heat drying treatment, nor the successive immersion steps, did not induce any morphological changes. Zooming in even further, the crystallinity of the AuNSs can be assessed as single-crystal nanospheres (Figure 2d).

**Figure 3:**
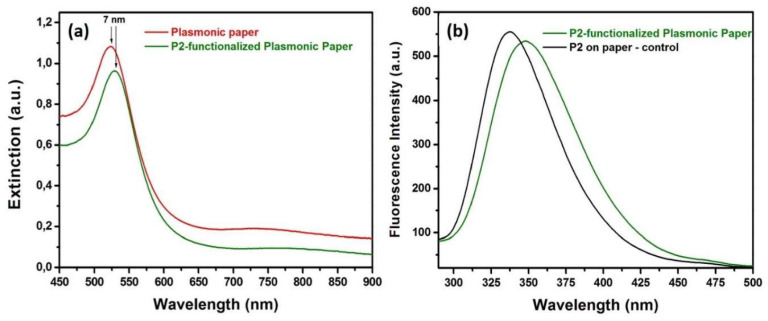
(**a**) The extinction spectra of the plasmonic paper before and after the functionalization with the P2 polypeptide and (**b**) fluorescence spectra of P2 on bare paper and on the designed plasmonic paper substrate.

After the adsorption of the AuNSs onto the paper matrix, the nanoplatform’s functionalization with the P2 polypeptide was further addressed. Considering the amino groups’ affinity to bind to the gold surface, the P2 was grafted by dropping 10 µL aqueous solution of 50 µM of P2 molecules to create a polypeptide monolayer on the plasmonic paper. After the functionalization with P2, the extinction band of the plasmonic paper records a 7 nm red-shift indicating that the micro-environment in the close vicinity of the AuNSs has changed again, thus confirming the successful functionalization with the P2 polypeptide (Figure 3a). Moreover, steady-state fluorescence technique was next employed to obtain valuable information regarding the electrostatic interaction between P2 peptides and AuNSs’ surface (Figure 3b), by monitoring the strong fluorescence emission of the P2-functionalized plasmonic paper (Figure 3b: green spectrum), compared to the free P2 molecules dropped onto Whatman paper (Figure 3b: black spectrum), within the spectral region between 295 and 500 nm, employing a fixed excitation wavelength at 280 nm. As we can see in Figure 3b: black spectrum, the P2 polypeptides grafted directly onto the Whatman paper exhibits a strong intrinsic fluorescence emission band at 339 nm, which originates from the emission of the Tryptophan residues. Tryptophan residues are, in general, highly sensitive to changes of the local environment, and, therefore, when the P2 molecules were grafted onto the plasmonic paper, a red-shift of the fluorescence emission up to 9 nm, from 339 to 348 nm, was noticed, indicating polarity changes around the tryptophan residues, which corroborate results obtained from the LSPR spectra, leading to the conclusion that the P2 polypeptide was successfully bound to the AuNSs adsorbed onto the paper substrate.

### 2.2. The Biocompatibility of the Functionalized Plasmonic Paper

**Figure 4:**
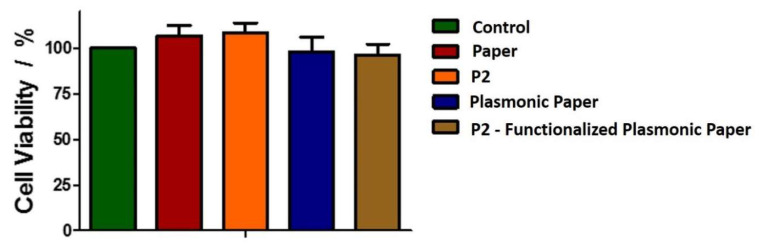
Biocompatibility of the new nanoplatforms against human BJ cells.

First, we evaluated the toxic effect against eukaryotic cells of the nanoplatforms, or the component materials (peptide alone and the Whatman paper itself) by performing MTT analysis, and calculating the % viability according to the described method in Section 3. The changes in the absorbance at 570 nm were monitored after their 24 h incubation with the proposed nanomaterials placed in direct contact with the cells, these modifications translate in changes in the BJ cells’ viability. To note that by placing the material onto the cell monolayer, the human BJ cells are in direct contact with our paper platform, and, consequently, we can successfully evaluate its effect on both cells viability as well as morphological alterations investigated through the fluorescence microscopy. The obtained cell viability results are presented in Figure 4. Compared to the control BJ cells, the cells grown in the presence of P2 or the paper alone present a slightly increased viability, thus demonstrating that the two components are not toxic for the skin cells, as expected. Previous studies have reported that, paper-based platforms, like the Whatman paper, are gaining ground in biomedical applications due to their biocompatibility with eukaryotic cells, cost efficiency, accessibility, etc. [22]. Moreover, these results are in good agreement with a previous study on the P2 polypeptide, which assessed that P2 has no toxicity against eukaryotic cells at the concentrations used herein [31].

However, in the presence of the plasmonic paper and P2-functionalized plasmonic substrates, a small decrease in cell viability is observed. After the incubation with both plasmonic systems, the viability decreases 10%. Nonetheless, the decrease is not significant, indicating that the new obtained plasmonic nanoplatforms are not toxic for human skin cells, favoring their further implementation in antibacterial applications.

### 2.3. Evaluation of the BJ Cells Morphology and Viability by In-Vitro Fluorescence Imaging

**Figure 5:**
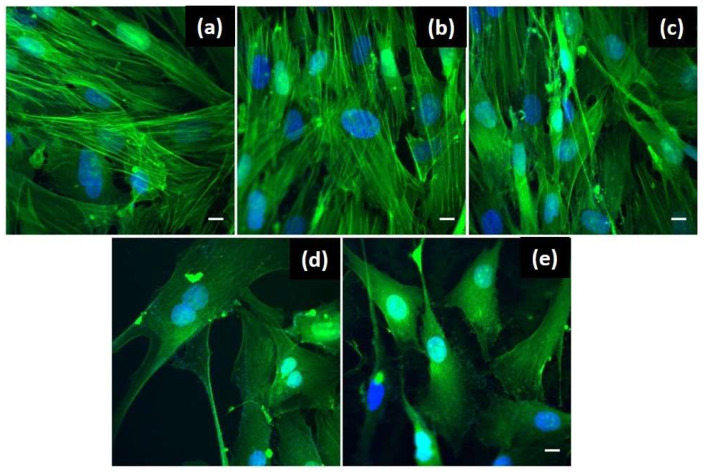
Confocal fluorescence microscopy images emphasizing the structural changes of the BJ cells after the 24 h treatment with: (**a**) no treatment-control cells; (**b**) Whatman paper itself; (**c**) free P2; (**d**) plasmonic paper-based nanoplatform and (**e**) P2-functionalized plasmonic nanoplatform. Scale bar is 10 μm.

Further, we investigated the structural changes induced in the BJ cells by the plasmonic nanoplatforms. Specifically, the cytoskeleton, which is responsible with maintaining the cell’s shape, help with cell/organelle movement or cell division, and the nucleus, which coordinates the cell activity, were monitored. Figure 5 presents representative confocal fluorescence microscopy images of the BJ cells in all tested conditions after the staining process of the nucleus with Hoechst 33,342 (blue) and actin filaments with Phalloidin-FITC (green). The control cells have an elongated, bipolar morphology, with actin filaments well-organized, almost parallel, going almost from one end to the other end of the cell (Figure 5a). Additionally, the nucleus shows with its specific ovoid morphology. Similar characteristics are observed for the cells grown in the presence of the Whatman paper (Figure 5b) and peptide P2 (Figure 5c), thus confirming, along with the viability tests, that alone the two do not induce any changes in the cell structure.

In the cases of the plasmonic paper-based nanoplatform without (Figure 5d) and functionalized with the antimicrobial peptide P2 (Figure 5e), some morphological changes are observed. Instead of the specific elongated shape, the BJ cells show an altered shape becoming smaller with a stellar morphology, with 3 or more processes extending from the cell body. Also, the actin filaments are less organized as compared with the control cells. Even though, the shape of the cells has changed, the nucleus doesn’t show any modifications, indicating that the cellular functions are not affected. This observation is sustained also by the fact that the cells were found in division in both cases. Anew, the obtained results are well correlated with the reported for MTT viability assay, the number of grown cells is decreased in the presence of the plasmonic nanoplatforms despite showing cell division functionality. To conclude, the developed plasmonic nanoplatforms were demonstrated to be biocompatible for the human skin cells using two complementary techniques, thus supporting their further testing in terms of antimicrobial activity.

### 2.4. Antimicrobial Activity on Planktonic Bacteria and Bacterial Biofilms

Further, the microbial activity of our plasmonic paper-based nanoplatform was tested in two different cases: (i) planktonic bacteria: generally described as independent, untethered planktonic cells in diluted suspensions, and (ii) bacterial biofilms: formed by the adhesion of the bacterial cells to each other or to a surface. For both situations, the Gram-positive *Staphylococcus aureus 12600* and Gram-negative *Escherichia coli 25922* strains were chosen for the validation and evaluation of the antimicrobial effect of the proposed plasmonic paper-based nanoplatform.

In the case of the planktonic bacterial suspensions prepared as described in Section 3, the plasmonic paper was investigated in two configurations, specifically in the absence and functionalized with the chemically synthesized P2 polypeptide. As control, we followed the same experimental procedure without the plasmonic paper.

Firstly, the classical dilution-extraction colony-counting method was employed. The plasmonic paper with and without P2 showed strong antimicrobial activity, as supported by their capacity to reduce the bacterial growth with more than 6 logCFU/mL. The residual antimicrobial activity of the discs combinations after extraction was absent, as checked by the disc diffusion method both towards *S. aureus* and *E. coli.* However, it seems that though the antimicrobial components are extractible, they are not as equally diffusible, because non-extracted discs did not develop large inhibition diameters (Table 1). An inhibition zone of 5 mm indicates the absence of an antimicrobial diffusible effective substance, as the 5 mm are represented by the disc diameter itself.

**Figure 6:**
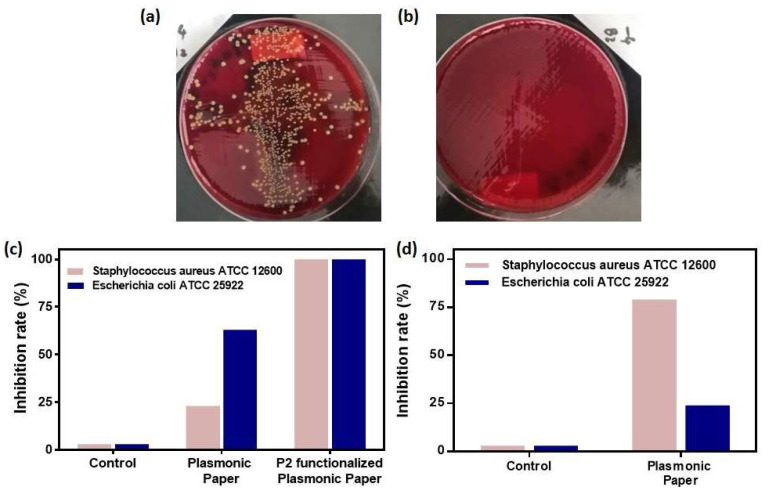
Representative digital images of the colony counting plates with *Staphylococcus aureus ATCC 12600* showing the anti-microbial activity against planktonic bacteria without (**a**) and with the P2-functionalized plasmonic paper (**b**); The bacterial growth inhibition rates for (**c**) planktonic bacteria and (**d**) bacterial biofilm determined for the as-designed paper-based nanoplatform when applied to both *Staphylococcus aureus ATCC 12600* and *Escherichia coli ATCC 25922* bacterial cultures.

Further, the differential antimicrobial activity of our designed plasmonic nanoplatform, as documented in the second, more sensitive colony counting experiment, can be visually confirmed by observing the colony counting plates as seen in Figure 6a,b, in the presence of the P2-functionalized plasmonic paper the bacterial growth is drastically reduced. To assess the capability of each paper-based nanoplatform to efficiently inhibit the bacterial growth, the number of colony forming units (CFU/mL) was determined and further expressed as percentual inhibition rates (Figure 6). With respect to the control groups, where the growth of the planktonic bacteria is undisturbed, the paper-based nanoplatform inhibits the growth of both strains with different efficiencies. For instance, the plasmonic paper alone inflicts a growth reduction of 23% (6 logCFU/mL) of the *Staphylococcus aureus 12600* bacteria, however, its anti-bacterial activity is much more efficient for *Escherichia coli 25922,* leading to a 63% (6.6 logCFU/mL) inhibition rate. After the functionalization of the plasmonic paper with the P2 polypeptide, the bacterial colony formation is totally inhibited, the growth reduction rate being significantly improved by reaching for both Gram-positive and Gram-negative strains 100% (7 logCFU/mL). Gold nanoparticles themselves have been proven to alter the bacterial surface by adhering to it, thus inducing a series of inhibition events leading to the loss of cellular integrity. Hence, they produce a rather small inhibition of *S. aureus* [27]. Furthermore, a similar paper-based nanoplatform functionalized with silver-coated gold nanoparticles show a gradually increasing inhibition rate of *E. coli* reaching 100% after 24 h [32]. These values are well-correlated with the obtained inhibition rates for our AuNSs P2-functionalized paper-based nanoplatform.

Furthermore, the bacterial growth inhibition was tested for the plasmonic paper during the biofilm formation of both bacterial strains. In this specific case, the bacterial cells were stained with the crystal violet dye. For the determinations of the inhibition rates, the optical densities at 500 nm were extracted for the control growth samples, as well as growth probes with the plasmonic paper. A correction was then applied to subtract the background contribution and the percentual equivalents were calculated. As expected, the control samples exhibit 0% inhibition, as their growth process is not altered or disturbed in any way. Compared to the antimicrobial activity against planktonic bacteria, for bacterial biofilm growth inhibition the plasmonic paper is 3.3-fold more efficient against *Staphylococcus aureus* ATCC 12600 reaching a 79% growth reduction rate compared to 24% against *Escherichia coli* ATCC 25922.

## 3. Materials and Methods

### 3.1. Chemicals

Hydrogen tetrachloroaurate-(III) trihydrate (HAuCl_4_ ∙ 3H_2_O, 99.99%), sodium borohydride (NaBH_4_), Hexadecyltrimethylammonium bromide (CTAB, 96%), Cetyltrimethylammonium chloride solution (CTAC), ascorbic acid (AA) and Whatman^®^ qualitative filter paper, Grade 1 (Whatman no. 1) were purchased from Sigma-Aldrich (St. Louis, MO, USA). The synthetic RRWHRWWRR-NH_2_ polypeptide (further noted as P2) was synthetized by Pierce Protein Biology, Thermo Fisher Scientific (Mt Prospect, IL, USA). All chemicals were of analytical grade, and all aqueous solutions were prepared using ultrapure water (resistivity~18 MΩ).

### 3.2. Colloidal Gold Nanospherical Synthesis

For the fabrication of the CTAC-stabilized gold nanospheres (AuNSs), an adapted version of the successive seed-mediated growth approach previously reported by Zheng et al. [33] was employed. The chemical synthesis method is based on two steps: (i) synthesis of initial CTAB-capped Au clusters by the addition of a NaBH_4_ solution to a mixture containing 0.25 mM HAuCl_4_ (as Au precursor) and 100 mM CTAB, and (ii) the generation of the CTAC-coated AuNSs from the Au clusters, which served as the initial seeds, by adding 10 µl of the as-synthesized Au clusters to a freshly prepared solution of 200 mM CTAC (serving as stabilizing agent) and AA (serving as reducing agents), followed by a 2 mL of 0.5 mM HAuCl_4_ solution. The final mixture was then left to react for 15 min at 27 °C leading to the formation of Au seeds having 10 nm in diameter. The as-prepared seeds were then purified by centrifugation at 14.500 RTM for 30 min using a Mikro 220R from Hettich (Westphalia, Germany) centrifuge and redispersion in 20 mM CTAC solution.

### 3.3. Fabrication and Functionalization of the Plasmonic Paper Support

In order to fabricate the plasmonic paper substrate, Whatman no.1 filter paper was employed, from which paper strips were cut. For the immobilization of the nanoparticles, the strips were then immersed in the colloidal solution and left to soak for 10 min. The substrates were dried at 45 °C for an additional 10 min. To ensure a high loading of AuNSs on the paper fibers, the immobilized nanoparticle concentration was increased by the application of the immersion procedure for 3 consecutive times. The plasmonic paper was then functionalized with synthetic P2 polypeptide, by dropping 10 µL aqueous solution of 50 µM of P2 molecules to create a polypeptide monolayer on the plasmonic paper. To note that Peptide P2 was synthetized and characterized in a previous study together with 7 other de novo short tryptophan- and arginine-rich peptides [31]. As reported, the 5 arginine residues (R) and the amidated C-terminus, give P2 a + 6 net charge, which will facilitate the electrostatic interaction with the negatively charged bacterial membrane. The peptide also has 3 tryptophan amino-acids (W), which facilitate the peptide insertion into lipid membranes. Based on previous results, P2, as well as peptides containing a RW pattern in their structure, have high antimicrobial properties. P2 exhibited MIC values between 5.2 and 25.6 µM against Gram positive (B. subtilis and S. aureus) and Gram negative bacteria (E. coli), with no hemolytic activity or cytotoxicity against lymphocytes at a concentration of 86.67 µM [31].

### 3.4. Cell Culture

Human foreskin cell line, BJ was purchased from the ATCC Cell Line Bank, cultured in Modified Eagle’s medium (MEM), and supplemented with 10% fetal bovine serum (FBS) and penicillin-streptomycin (1%−100 units/mL), in a humidified atmosphere of 95% air/5% CO_2_ at 37 °C. All cell cultivation media and reagents were purchased from Biochrom AG (Berlin, Germany).

The structural changes induced in the BJ cells by the paper support itself, AuNSs-immobilized and, subsequently, P2-functionalized plasmonic paper were investigated using fluorescence microscopy. For fluorescence imaging, the actin filaments were stained using Phalloidin-FITC (Sigma-Aldrich, St. Louis, MO, USA), while, for the nucleus, Hoechst 33342 (Invitrogen, Waltham, MA, USA.) was used, as briefly described. First, the BJ cells were grown on circular coverslips placed in 24 well plates at a density of 20,000 cells/well and grown for 24 h. Further, sterile discs having a diameter of 5 mm were cut from the bare Whatman paper (as control), AuNSs immobilized and P2-functionalized plasmonic substrates and placed inside the wells for an additional 24 h treatment. To note, that P2 alone was tested as well, and untreated cells were considered negative control. After the desired treatment time, the cells were washed with PBS and formaldehyde-fixed (PFA 4%). After this, the cells were washed again with PBS and permeabilized with Triton X-100 in PBS (0.1%). Then, an additional washing step was performed using PBS, followed by the permeabilization with Triton X-100 (0.1% in PBS). Subsequently, the treated BJ cells were washed again, then incubated with the two fluorescent dyes, in the dark, at room temperature for an hour and a half. Finally, the cells were washed with PBS and fixed with FluorSaveTM (Merck KGaA, Darmstadt, Germany).

### 3.5. In-Vitro Cell Viability Assay

The cell viability was assessed using a MTT assay, as briefly described [34]. First, the BJ cells were plated into 24 well plates in similar conditions as mentioned above. After 24h of growing the cells in the presence of the nanoplatforms, the medium and the discs were removed from the wells, which were then incubated with a final concentration of 1 mg/mL of MTT. After 4 h, the medium was removed and DMSO was added to dissolve the formed crystals. The optical absorbance was recorded at 570 nm using the plate reader Mithras LB 940 (Berthold, Bad Wildbad, Germany) and the absorbance values of blank wells (only DMSO) were extracted in order to calculate the cell viability using the expression:

(1) $$\%\;viable\;cells=\frac{Corrected\;absorbance\;of\;treated\;cells}{Corrected\;absorbance\;of\;control\;cells}\times 1$$

### 3.6. Bacterial Strains and Growth Conditions

For the antimicrobial activity assessment, two reference bacterial strains were tested, specifically the Gram-positive *Staphylococcus aureus ATCC 12600* and Gram-negative *Escherichia coli ATCC 25922* strains. The cultures of both strains were obtained by their incubation in Mueller-Hinton broth (Oxoid CM0405 Lot 2216266) for 2 h. The optical density of bacterial suspensions was calibrated spectrophotometrically at 0.5 McFarland standard equivalence and adjusted afterwards according to the requirements of each experimental method.

### 3.7. Antimicrobial Activity on Planktonic Bacteria

Prior to the antimicrobial tests, the plasmonic nanoplatforms were cut into discs having a diameter of 5 mm, which were then sterilised by the exposure to UV light for 15 min on each side. In order to evaluate the anti-bacterial activity of the plasmonic paper-based nanoplatform, half of the discs were implemented after the sterilization, while, for the assessment of the cumulated/combined activity with the P2 antimicrobial polypeptide, the other half was functionalized with a 50 µM P2 polypeptide solution and allowed to dry at 36 ± 1 °C for 1 h.

The colony-counting method was used firstly in a classical dilution-extraction variant. Using a 96 wells round bottom microtitration plate (M220 24A bioMerieux, Lot 1084), a quantity of 100 µL Mueller-Hinton broth per well was distributed in order to extract the antimicrobial soluble components, each disc being introduced in a corresponding well. Broth sterility and bacterial growth controls were added. After incubation for 24 h at 36 ± 1 °C, the discs were removed from the microtiter wells using sterile forceps and then 10 µL from a 10^−2^ dilution of a 0.5 McFarland bacterial suspension were added in all corresponding wells, except broth sterility and control well. After 24 h incubation at 36 ± 1 °C, 1 μL from each well was inoculated on blood agar in order to evaluate the bacterial growth.

Separately, the plasmonic paper discs with and without P2 after extraction as well as unsubmitted discs were distributed onto Mueller-Hinton agar inoculated with a 0.5 McFarland bacterial suspension for assessing the remaining antimicrobial activity using the disc diffusion method.

Furthermore, in order to substantiate the contribution of each component of our P2-functionalized paper-based nanoplatform to the antimicrobial activity, the bacterial cultures were calibrated at a turbidity equivalent with 0.5 McFarland standard and diluted to a final concentration of~10^2^ UFC/mL. 1 mL of the *Staphylococcus aureus* and *Escherichia coli* diluted suspensions were placed in each of a series of 25 mL sterile glass tubes. The plasmonic discs with and without the P2 polypeptide were immersed in the bacterial suspension and maintained at 36 ± 1 °C in a shaking incubator for 24 h. After the incubation, 50 µL of each probe were extracted and diluted by factors ranging from 10^−1^ to 10^−5^. The obtained probes were inoculated on Columbia blood agar plates (Columbia agar OXOID CM 0331, Lot 2377465 added with 7% *v/v* sheep blood provided by Cantacuzino Institute Animalery) and incubated at 36 ± 1 °C for 24 h. Broth sterility control and bacterial growth control were also prepared in the same experimental conditions.

After the treatment, the plates with the appropriate dilution rate, which lead to isolated colonies, were further selected. The number of colony forming units (CFU) per milliliter was determined according to the equation:

N = *n* × V × D (2)

where *n* = the number of colonies counted on the plate, V = volume factor and D = dilution factor.

The growth reduction percent was calculated for all samples after the exposure to both *Staphylococcus aureus* and *Escherichia coli* using the following expression:

(3) % growth reduction=Nr. of CFUml in the control tube−Nr. of CFUml in the treated tubeNr.of CFUml in the control tube×100

The antimicrobial activity was determined in triplicate for both treated with the plasmonic paper-based nanoplatform with and without P2, and control group samples.

### 3.8. Antimicrobial Activity on Bacterial Biofilms

The determination of the antimicrobial activity on biofilms was performed on static microplate biofilm assays. The plasmonic nanoplatform discs were placed in round bottom microplate (M220 24A bioMerieux, Lot 1084) wells containing bacterial suspension calibrated at the turbidity of 0.5 McFarland standard diluted at 10^−3^ dilution, and incubated at 36 ± 1 °C for 24 h. After the incubation, the content of the wells was removed, and the wells were thoroughly washed with sterile physiologic saline solution. The obtained biofilm was then fixed with 150 μL anhydrous methanol for analysis (Merck KGaA CAS No 67-56-1) for 5 min followed by the staining process with 1% Gram Crystal Violet (Biognost GC1-OT-250) for an additional 20 min interval before performing an additional plate washing step with water. Next, the fixed dye was solubilized with a 33% glacial acetic acid solution for analysis (Chimreactiv S.R.L. CAS 64-19-7) and the optical density (OD) was measured at a wavelength of 500 nm. Microplate wells without the plasmonic nanoplatform were prepared in the same experimental conditions as control samples. The experiment was performed in triplicate for each probe.

The reduction percent of the biofilm formation for *Staphylococcus aureus* and *Escherichia coli* was calculated using the following expression:

(4) $$\%\;reduction=\frac{Corrected\;OD\;of\;the\;control\;well-Corrected\;OD\;of\;the\;treated\;well}{Corrected\;OD\;of\;the\;control\;well}\times 100$$

The correction was applied by subtracting the ODc, which stands for the mean of the 3 ODs of the negative controls plus 3 times the negative control standard deviation, from both of the ODs of the control and treated growth wells.

### 3.9. Characterization Methods

The UV-Vis extinction spectra of the colloidal AuNSs were recorded using a Jasco V-670 double-beam UV-Vis-NIR spectrophotometer (from Jasco International CO., Ltd. (Tokyo, Japan), with a 2 nm bandwidth and 1 nm spectral resolution. The recorded spectra were analysed with the Spectra Manager software. The size and morphology of the synthesized AuNSs in aqueous solution were then examined using a FEI Tecnai F20 field emission Transmission Electron Microscope (TEM), operating at an accelerating voltage of 200 kV and equipped with Eagle 4k CCD camera. The colloid was added dropwise onto a carbon film covered copper grid for TEM analyses. Dynamic light scattering (DLS) and Zeta Potential measurements of the colloidal AuBPs were performed using a Nano ZS90 Zetasizer analyzer from Malvern Instruments equipped with a He-Ne laser (633 nm, 5 mW). The used analysis parameters were a scattering angle of 90° and temperature of 25 °C. All samples were measured three times and the mean value has been reported.

After the controlled immobilization of the AuNSs on the Whatman paper, the plasmonic responses of the new as-formed plasmonic nanoplatforms were collected using a portable Ocean Optics USB 4000 optical UV-Vis spectrophotometer coupled to a ZEISS Axio Observer Z1 inverted microscope with 10× ZEISS objective (NA = 0.45) through an optical fiber with a core diameter of 600 μm. The extinction spectra were recorded in absorption mode, using 5 accumulations and 50 milliseconds integration time, the spectral resolution of the spectrophotometer being 0.2 nm. Subsequently, the morphology and the uniformity of the new nanoplatforms were investigated by Scanning Electron Microscopy (SEM) using a FEI Quanta 3D FEG dual beam scanning electron microscope operating at an accelerating voltage of 30 kV. The plasmonic paper were sputtered using a Q150R ES automatic Sputter Coater, in an argon atmosphere, with 5 nm gold layer for 10 min prior to the SEM investigation in order to inhibit charging, reduce thermal damage and improve the secondary electron signal required for topographic examination in the SEM. High Resolution TEM (HR-TEM) images were then recorded using a Jeol 2010F electron microscope working at 200 kV. For the HRTEM observation, the paper-based plasmonic nanoplatforms were wetted with alcohol, then scratched with a scalpel, and the debris was suspended in alcohol using ultra-sonication for 15 min to disperse it. A droplet of the solution was dribbled onto a holey carbon grid 300 mesh microscopy grid and allowed to dry.

Fluorescence emission measurements were collected at room temperature using for Jasco LP-6500 spectrofluorometer containing an epifluorescence accessory (EFA 383 module) with a 1 nm spectral resolution, and equipped with a DC-powered 150W Xenon lamp as excitation source. The excitation and emission bandwidths were fixed at 3 nm. Fluorescence spectra were recorded in the wavelength range of 290–500 nm, employing a fixed excitation wavelength at 280 nm.

In-vitro fluorescence images were taken using a confocal microscope (Andor DSD2 Confocal Unit) mounted on an epifluorescence microscope, Olympus BX-51. Nucleus images were recorded using an appropriate DAPI/Hoechst filter cube (excitation filter 390/40 m, dichroic mirror 405 nm and emission filter 452/45 nm) and the actin filaments were evidenced using a GFP/FITC filter cube (excitation filter 466/40 nm, dichroic mirror 488 nm and emission filter 525/54 nm). Images were further processed using the ImageJ software.

## 4. Conclusions

In conclusion, in this paper, we synergistically combined the advantage of the positively-charged gold nanospheres electrostatically immobilized onto a Whatman paper, as miniaturized plasmonic transducers, with the synthetic RRWHRWWRR-NH_2_ polypeptide, as potent antimicrobial peptide, to obtain an efficient nanoplatform able to inhibit both the microbial activity and biofilm formation of two reference bacterial strains: *Staphylococcus aureus ATCC 12600* and *Escherichia coli ATCC 25922*, respectively. Specifically, after the nanoparticles’ loading onto the negatively-charged cellulose fibres, as a result of an easy immersion approach, the as-engineered plasmonic paper was optically and morphologically characterized to prove the well-conserved optical response, as well as the uniform distribution of the nanoparticles onto the 3D flexible paper fibre scaffold. Finally, the antimicrobial activity of the grafted P2 peptide onto the plasmonic paper was proved to be significantly enhanced, namely 100%, against both microorganisms tested. Moreover, the plasmonic paper significantly reduced the in-vitro biofilm formation of *Staphylococcus aureus* up to 79% and *Escherichia coli* up to 24% compared to without plasmonic paper. Our functionalized plasmonic paper-based antimicrobial nanoplatform relies on a simple and cheap fabrication method which integrates biocompatibility features and highly efficient anti-microbial activity, thus becoming a good candidate for further use as an antimicrobial nanoplatform.

## Figures and Tables

**Table 1:** Comparative antimicrobial activity of the different disc combinations before and after extraction of antimicrobial active components expressed by the diameter of inhibition.

Disc/Peptide	*Staphylococcus aureus 12600*		*Escherichia coli 25922*	
	Before Extraction	After Extraction	Before Extraction	After Extraction
Blank/P2 50 mM	5 mm	5 mm	5 mm	5 mm
Plasmonic Paper/0	6 mm	5 mm	6 mm	5 mm
Plasmonic Paper/P2 50 mM	6 mm	5 mm	6 mm	5 mm
